# Robot-supported assessment of balance in standing and walking

**DOI:** 10.1186/s12984-017-0273-7

**Published:** 2017-08-14

**Authors:** Camila Shirota, Edwin van Asseldonk, Zlatko Matjačić, Heike Vallery, Pierre Barralon, Serena Maggioni, Jaap H. Buurke, Jan F. Veneman

**Affiliations:** 1 Rehabilitation Engineering Lab, Institute of Robotics and Intelligent Systems, Department of Health Sciences and Technology, ETH Zürich, Lengghalde 5, 8092 Zürich, Switzerland; 2 Department of Biomechanical Engineering, MIRA, University of Twente, Drienerlolaan 5, 7522 NB Enschede, The Netherlands; 3 University Rehabilitation Institute, Republic of Slovenia, Linhartova 51, SI-1000 Ljubljana, Slovenia; 4 Faculty of Mechanical, Maritime and Materials Engineering, Delft University of Technology, Mekelweg 2, 2628 CD Delft, The Netherlands; 5 Health Division, Tecnalia Research and Innovation, Paseo Mikeletegi 1, 20009 Donostia-San Sebastian, Spain; 6 Sensory-Motor Systems Lab, Institute of Robotics and Intelligent Systems, Department of Health Sciences and Technology, ETH Zürich, Sonneggstrasse 3, 8092 Zürich, Switzerland; 7 Hocoma AG, Industriestrasse 4a, 8604 Volketswil, Switzerland; 8 Roessingh Research and Development, Roessinghsbleekweg 33b, 7522 AH Enschede, The Netherlands

**Keywords:** Standing, Walking, Balance performance, Assessment, Rehabilitation robots, Posturography

## Abstract

Clinically useful and efficient assessment of balance during standing and walking is especially challenging in patients with neurological disorders. However, rehabilitation robots could facilitate assessment procedures and improve their clinical value. We present a short overview of balance assessment in clinical practice and in posturography. Based on this overview, we evaluate the potential use of robotic tools for such assessment. The novelty and assumed main benefits of using robots for assessment are their ability to assess ‘severely affected’ patients by providing assistance-as-needed, as well as to provide consistent perturbations during standing and walking while measuring the patient’s reactions. We provide a classification of robotic devices on three aspects relevant to their potential application for balance assessment: 1) how the device interacts with the body, 2) in what sense the device is mobile, and 3) on what surface the person stands or walks when using the device. As examples, nine types of robotic devices are described, classified and evaluated for their suitability for balance assessment. Two example cases of robotic assessments based on perturbations during walking are presented. We conclude that robotic devices are promising and can become useful and relevant tools for assessment of balance in patients with neurological disorders, both in research and in clinical use. Robotic assessment holds the promise to provide increasingly detailed assessment that allows to individually tailor rehabilitation training, which may eventually improve training effectiveness.

## Background

This work was developed in the frame of the project “STate of the Art Robot-Supported assessments” or STARS, as part of the COST Action TD1006 “European Network on Robotics for NeuroRehabilitation” [1]. STARS is intended to equally serve clinical practitioners, technology developers and manufacturers, as well as researchers and scientists active in the field of neurorehabilitation. The goal is to give recommendations for development, implementation, and administration of different indices of robotic assessments, grounded in the scientific literature available at this time. ‘Robotic’ or ‘robot-supported’ assessment here points to quantitative assessment performed through the use of specific robotic tools, for example rehabilitation robots or robots especially developed for assessment.

Maintaining balance is a critical component of many daily tasks, from standing upright to walking on uneven terrain. Here, we define balance as the continuous and adequate adaptation of body posture to avoid falling. Impaired balance is common in patients with diverse health conditions, in particular those with neurological damage through, e.g., cerebral vascular accidents (CVA, or stroke), traumatic brain injuries (TBI) or spinal cord injuries (SCI) [2]. In these patients, impaired balance manifests itself as a reduction of functional abilities, i.e., difficulty in sitting, standing or walking [3], as well as in transitions such as sit-to-stand, stand-to-walk or turning. Thus, restoring balance in these populations is critical to improve patients’ quality-of-life and return to society.

Despite its importance, the assessment of balance in clinical practice remains rather crude and has limited value in the treatment of patients with neurological disorders. Outside of a few specific contexts (e.g., vestibular patients), current assessments consist of qualitative scores of performance (e.g., normal, severely impaired) or measures of overall performance (e.g., completion time) in functional activities. Although many assessments are reliable (i.e., produce stable and consistent results) and have good fall-prediction validity [4], they provide limited information towards analysis or understanding of reduced performance. Further, there are very few assessments for patients that require support (e.g., canes, walkers), despite their remaining or potential abilities. This limits the richness and clinical value of balance assessments, e.g., balance assessments currently hardly help the clinician to tailor training to individual needs.

A challenge in better assessing balance is that it involves many underlying neuro-musculoskeletal and sensory body functions [5–7]. The relative importance of the involved body functions can vary across activities, and constraints or deficits in any of these body functions can impair or affect balance [8]. Neurological impairments generally affect multiple body functions simultaneously and, together with the development of compensatory strategies, obscures the relationship between specific impairments and outcomes of functional performance assessments.

The human ability to maintain balance is a complex function, and can be analysed from multiple viewpoints. Three are of particular interest here: i) static vs. dynamic balance; ii) the ability to maintain steady-state balance when facing different challenges; and iii) core strategies to maintain balance. At the highest, contextual level, balance can be separated between static and dynamic, depending on whether it is intended to maintain a body posture or avoid falls during movements like locomotion. Next, steady-state, anticipatory and reactive balance control are related to the types of challenge to balance that are counteracted [9]. These challenges can be further classified as internal or self-generated disturbances, such as voluntary reaching, and external, such as pushes [10]. Finally, balance control is achieved through movements that are composed of core strategies, or fundamental coordinated actions of the lower limbs intended to maintain or recover balance. For example, standing balance is maintained using the ankle, hip and stepping strategies, while walking balance uses foot placement strategies. Assessments can address different aspects of balance, according to these distinctions, e.g., i) assess during standing or walking, ii) assess with or without external perturbations or anticipated motions, iii) assess with procedures that require and measure specific balance strategies. To improve balance rehabilitation, it is critical to understand how neurological impairments have affected the different components of balance in a specific patient. It is thus critical to assess balance covering different aspects of the indicated spectrum [11, 12] to adequately measure its progress or deterioration, analyse the determinants of poor performance, and to personalize training.

New technologies could improve balance assessments by increasing information richness, precision and ease of procedures, and by expanding the range of tasks. In recent years, ‘advanced rehabilitation technology’, such as wearable sensors and rehabilitation robots, is being developed. Rehabilitation robots are devices that directly interact mechanically with the user, and can move their limbs or support their body posture through use of robotic technology. Regarding assessment of balance, these developments may have the following benefits:

- faster and more repeatable procedures and assessment results, through automatic acquisition and processing of sensor data, instead of subjective observation and classification;
- improved assessment of “severely affected” patients, by measuring the amount of assistance provided during task execution in patients that cannot perform a task on own effort;
- improved assessment of reactive and dynamic balance, through well-defined perturbations, also during locomotion;
- improved information richness of assessments through procedures and measures that relate to determinants of poor functioning; and
- combined training and assessment by use of the same devices for both procedures, especially in cases where patients require functional support to accomplish a task.

Despite the introduction of rehabilitation robots into clinical practice [13], accessibility – including financial costs and reimbursement models – and familiarity of these devices are still barriers to their widespread use in the clinic.

In this paper, we present an overview of the potential use of emerging robotic devices in the assessment of balance. We propose a classification of these devices, and specify requirements for these technologies to be useful for assessment of balance. This paper focuses on balance assessments and rehabilitation in stroke, and includes both static and dynamic balance, especially during standing and level-ground walking. Two experimental robotic devices specifically developed to perform assessment of balance are presented as example cases of robotic assessment. We finalize by highlighting current challenges and recommendations towards the adoption of robotic devices in clinical assessment of balance.

## Clinical practice and developments in assessment of balance

In contrast to ‘diagnostics’, which investigates and determines the (physical) damage, abnormality or diseased state of the body, ‘assessment’ measures the (deterioration of) functional performance related to specific tasks in the context of such damage, abnormality or disease. This section will describe the main approaches to balance assessment, and identify shortcomings.

### Clinical assessments trade off information richness and duration of the assessments

In current stroke rehabilitation, the assessment of balance relies on various well-accepted clinical tests. These clinical tests generally start from an overall (high-level) functional perspective, and score a patient’s ability to perform specific activities while maintaining balance using a classification based on therapist observation, or simple measures related to task completion (e.g., completion time). Important examples are listed in Table 1. Extensive reviews and more detailed descriptions of clinical assessment procedures can be found in [5, 14, 15].

**Table 1:** Overview of several widely used clinical assessments for balance function

Clinical assessment	Type of balance assessed through procedure						Scoring
	Steady state		Anticipatory		Reactive		
	S	W	S	W	S	W	[S = Standing / W = Walking]
Romberg test [77]	X	-	-	-	-	-	Ability to stand with eyes closed compared to eyes opened: able/unable, or time (in seconds) position was maintained.
One-leg stance test, or single leg support, or timed unipedal stance test [78]	X	-	-	-	-	-	Time in seconds until one-leg stand is ended, by: lowering the elevated foot on the floor, taking hands off the hip or touching the standing leg with the elevated foot.
Functional reach test [79]	X	-	X	-	-	-	Maximum distance reached (from start) in centimeters.
Lateral reach test [80]	X	-	X	-	-	-	Maximal lateral reach to the right and left (from start) in centimeters.
Get Up and Go test [81]	-	-	X	X	-	-	Score from 1 (normal) to 5 (severely abnormal) based on perceived (ab) normality.
Timed Up and Go test [82]	-	-	X	X	-	-	Time (in seconds) to complete task and score from 1 to 5 based on observer’s perception of risk of falling.
Performance-Oriented Mobility Assessment (POMA), or Tinetti test [83]	X	X	X	X	X	-	Score from 0 (unable or highly impaired) to 1 or 2 (independent) on multiple tasks, based on ability to perform task and need of support (balance), or quality of movements (gait).
Berg Balance Test (BBT) [84]	X	-	X	-	-	-	Score from 0 (low) to 4 (high), based on ability to perform multiple separate tasks.
Balance Evaluation Systems test (BESTest) [11]	X	X	X	X	X	-	Score from 0 (severe impairment) to 3 (no impairment) on multiple tasks, based on ability to perform task; some related to time or speed.

Balance is separated into types according to two aspects: static (standing) or dynamic (walking); and steady-state, anticipatory or reactive (as defined in the Introduction). Scoring of performance in each assessment is also briefly described. (For general reference [15, 85])

Assessments like the TUG assume that overall task performance reflects the underlying quality of balance, but only indirectly measure balance performance. Typically, a single score that reflects performance (e.g., time to stand-up, walk a specific distance, turn, walk back and sit down) is measured; such metrics are one-dimensional, and provide little information towards understanding the components of poor balance performance and consequently for tailoring of the rehabilitation training. Other assessments, like the BesTest, score performance on a number of functions to more directly assess different ‘sub-systems’: Biomechanical Constraints, Stability Limits, Postural Responses, Anticipatory Postural Adjustments, Sensory Orientation, and Dynamic Balance during Gait. Such assessments provide multi-dimensional information and can provide more insight on the different causes and components of poor functional balance performance. However, the more dimensions are assessed, the more time is required for administering the assessment, which is a barrier for practical clinical use. In general, all clinical assessment procedures require a skilled clinician and typically at least half an hour of testing time, and include observation-based classification of the quality of performance on ordinal scales. Furthermore, none of the procedures evaluates reactive balance control during walking (see Table 1). This function is likely strongly correlated to the causes of many falls. This is an additional example of clinical assessments providing only limited information on the determinants of reduced balance performance.

### Instrumented assessments are quantitative and time-efficient, but have limited scope

To make assessments faster and less dependent on clinician skills, methods have been developed to perform quantitative, instrumented balance assessments, mainly following two approaches.

**Fig. 1:**
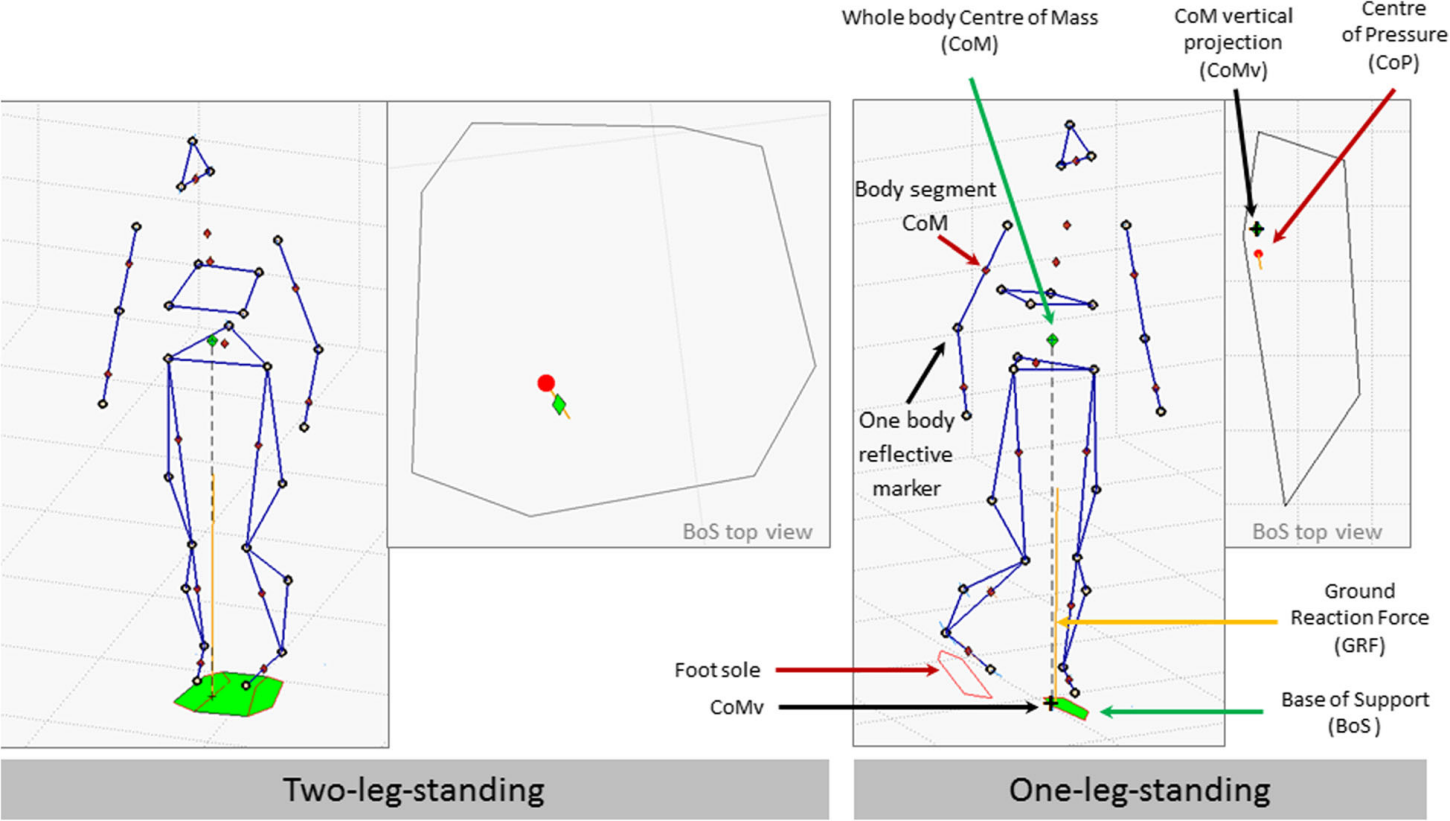
Illustration of the widely used biomechanical indicators (COM, COMv, COP, BOS, GRF) describing, or containing information regarding balance conditions. Features of one or a combination of these indicators is/are used to describe balance performance in current posturography and can be used in robotic assessment. More details on metrics based on such indicators can be found in the Appendix

A first instrumented approach is posturography, which evaluates postural control in standardized, instrument-based procedures. Posturography quantifies postural balance performance in either unperturbed or perturbed conditions during standing on a fixed or actuated instrumented platform. Posturography measures the ability to maintain the body’s Center of Mass (COM, or rather its vertical projection on the standing surface, COMv) within the Base of Support (BOS) (Fig. 1), which is a formal, physical definition of static balance. Center of Pressure (COP) motions reflect the subject’s active control to keep the body’s COMv within the BOS, and thus provide related but complementary information. Additional information on metrics used in posturography can be found in the Appendix. Comprehensive reviews on posturography can be found in [16–18].

Posturographic results are quantitative and have been shown to correlate with risk of falling or with some of the clinical balance assessments described above; however, the exact understanding of normality and abnormality, as well as the interpretation and inter-relation of the different metrics, remain a topic of research [18]. Posturography is, by its concept, limited to assessing balance performance during standing, and results obtained provide limited information on balance during other tasks, such as walking. This is supported by the observation that posturographic metrics correlate differently with different clinical scales, and sometimes not at all.

A second, currently more exploratory, approach is to equip subjects with unobtrusive sensors than can be worn during clinical procedures [19], or even during daily life [20] and calculate features from the collected data that may reflect balance performance or changes in performance [21, 22]. This can be considered as a data mining approach. In the field of stroke rehabilitation, some features have been shown to correlate with clinical metrics [23]. However, this approach is still in its infancy.

Concluding, even though several procedures of qualitative and quantitative assessment and measurement of balance in impaired subjects are used in clinical and research practice, there is still ongoing scientific debate on understanding human balance control and optimizing assessment methods and metrics. Clinical assessments mostly assess the overall functional performance, but do not address determinants or components of poor performance. Very few procedures consider reactive balance control. Posturography introduces quantitative assessment of balance during stance, and can include reactive balance. None of the established clinical or posturographic assessments include reactive balance control during walking.

## Balance assessment using robotic devices extends posturography

In recent decades, robotic devices for neurorehabilitation training of lower extremity functions have been introduced in clinical centers and research is being performed on their practical benefits for training and assessment [24–30]. Here, ‘robotic’ refers to any electromechanical device that uses actuators as part of a sensor-based control loop. A classification of the diverse robotic tools is presented in the next section. Different robotic tools can be used to provide precisely timed and sized, repeatable perturbations. Sensors are embedded in or can be easily added to robotic devices, enabling the use of a single device for therapy and assessment. The sensors could provide detailed measurement of balance-related reactions to specific perturbations. These data contain rich information about the determinants of reduced balance performance, and can potentially enrich the assessment to support personalized analysis and training.

Assessments through robot perturbations can be seen as an extension of ‘classical’ posturography, as they can be based on the same or analogous measures in different contexts. Robots can provide different types of perturbations, such as trips and hip shoves, as well as during different activities, especially during walking. These new technical possibilities may largely expand the ‘toolkit’ of posturography, and should be properly integrated into the field in order to understand how robotic assessments relate to, can contribute to, and can benefit from the extensive body of knowledge built up in the field of posturography.

Additionally, most types of robotic devices used in rehabilitation may be used to support patients in the execution of tasks. This could facilitate assessment in severely affected patients, i.e., those that are not able to perform the assessment tasks on their own effort (e.g., not able to stand or walk), but that have remaining functionality when provided with sufficient assistance (assistance-as-needed, AAN) [31]. Further, although most robots were developed for therapy, the sensors required for their operation continuously provide measurements that could be used to assess patients’ balance performance - such as joint angles, or applied forces. Measures during therapy activities, or short standardized protocols at, for example, the beginning and end of each therapy session, could provide more detailed information about patient progress as well as inform the effectiveness of different therapies.

A general concern for balance assessment with robotic devices is that the robot should not (excessively) influence or restrict the natural movement capabilities of the patients. The robot should minimally affect the baseline condition (e.g., walking), as well as patients’ reactions to perturbations. This is determined by the device’s degrees–of-freedom and their zero-force or transparency control performance [32]. Transparency, in this context, is understood as control methods that allow unhindered motion of the subject. The effect of blocking or adding substantial inertia to the human’s degrees-of-freedom involved in balance control should be carefully evaluated on their influence on natural execution of tasks. Several publications have assessed such aspects in the context of rehabilitation robots [33]. When using AAN, the robotic device should assist, but not completely execute, the task [31].

### Proposed classification scheme for rehabilitation robots for standing and walking

In this section, we provide a classification of robot types, structured according to characteristics that are relevant for possibilities and limitations for the assessment of balance. Within this classification, we position different robots that currently can be found in research or clinical practise. We only consider systems that allow execution of standing and walking functions by patients; thus, devices that only provide gait-like motions to the legs while being seated, such as those as classified as “Stationary Gait Trainers” in [27], are not included as they do not require any standing or walking balance capabilities from the patient.

We propose to classify rehabilitation robotic devices considering three important factors:

1. **Interaction** – how the device interacts with the body. We distinguish three main types of interaction:

s. **S**urface – if the device interacts by moving the surface on which the patient is standing or walking, such as perturbation platforms, treadmills, or actuated footplates;
c. **C**onnector – if the device interacts through a connection at a specific location on the body, e.g., at the pelvis, or through a harness; and
d. **D**istributed – if the device is connected to multiple locations on the body, such as in exoskeletons.

2. **Mobility** – how mobile the device is. We distinguish three main types of device mobility:

w. **W**earable – if the weight of the device is carried by the patient, as a device that is worn on the body, such as an exo-suit;
m. **M**obile – if the device mostly supports its own weight and can move in the environment (for example through wheels, stepping, or an overhead suspension); and
f. **F**ixed – if the device supports its own weight and cannot move around in the environment.

3. **Surface** – on what kind of surface the person stands or walks when using the device. We distinguish three main types of operation:

o. **O**ver-ground – if the device is operated with the patient standing or walking on a regular floor surface;
t. **T**readmill – if the device is operated with the patient walking (or standing) on a treadmill; and
p. **P**lates – if the device is operated with the patient standing or walking on an actuated plate that is continuously in contact with the feet (platform), or with each foot separately (footplates).

### Classification of sample robotic devices used in neurorehabilitation and their use for assessment

To demonstrate the use of the classification scheme provided in the previous section, we will give an overview of nine different types of robotic devices used or being developed in neurorehabilitation research, and classify them according to the scheme. The classification scheme can be applied to any type of robotic device for balance training and assessment; however, the nine types described include only currently existing configurations. Typical examples of these nine types, together with their classification and their potential abilities to assess balance, are presented in Table 2.

**Table 2:**
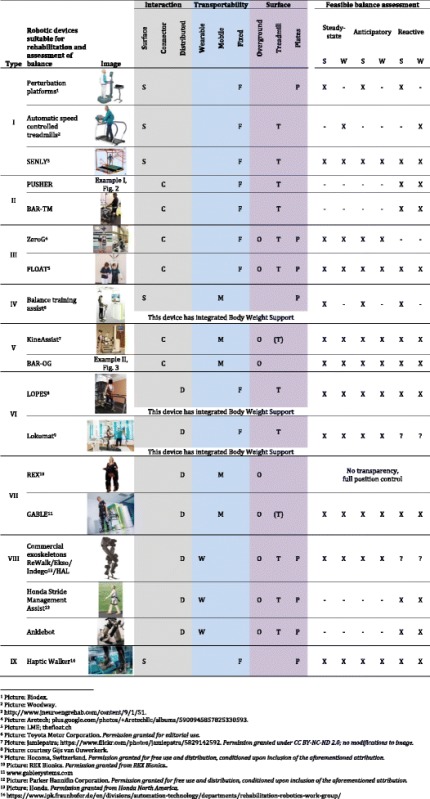
Examples of robotic devices used in rehabilitation that are suitable for balance assessment

Each device is listed by its rehabilitation robot type according to the classification introduced in this paper. The types of balance that can be assessed with each device are indicated in the last column (X – feasible to be assessed,? – unclear if feasible, because unknown how well the transparency control functions; S – Standing, W- Walking). For more details on specific devices, refer to section in the main text on “Classification of sample robotic devices used in neurorehabilitation and their use for assessment”.

#### Perturbation platforms and treadmills (SFP/SFT)

Perturbation platforms, e.g., actuated standing surfaces, are often used in ‘classical’ posturographic measurements [17]. Depending on the design, the standing surface can be moved in at least 1, and in up to 6, degrees-of-freedom. These robots are classified here as surface-, fixed-, plates-type (SFP) robots. They provide a controlled environment to challenge standing [34] and, if a treadmill is mounted on the platform, also during walking. Fast accelerations of such platforms can provide perturbations required for balance assessment, including stepping reactions [35]. Perturbation platforms are not suitable to provide assistance to patients.

Treadmills with high dynamic capabilities can also be considered perturbation platforms [36] when providing short acceleration or deceleration pulses, analogous to actuated platforms, although treadmills are typically limited to one perturbation direction. In this case, they are classified here as surface-, fixed-, treadmill-type (SFT) robots. Some treadmills use a separate belt for each foot (split-belt treadmills), allowing different perturbation directions on each side. Treadmills that can move in the two planar directions (anterior-posterior and medial-lateral), as well as in the orientation of the walking surface, are sometimes named OmniDirectional Treadmills. As an example of this treadmill category, the SENLY research platform [37] consists of two separate treadmills with additional actuators that allow perturbations to the feet both in the anterior-posterior and medial-lateral directions.

#### Treadmill-mounted pusher devices (CFT)

**Fig. 2:**
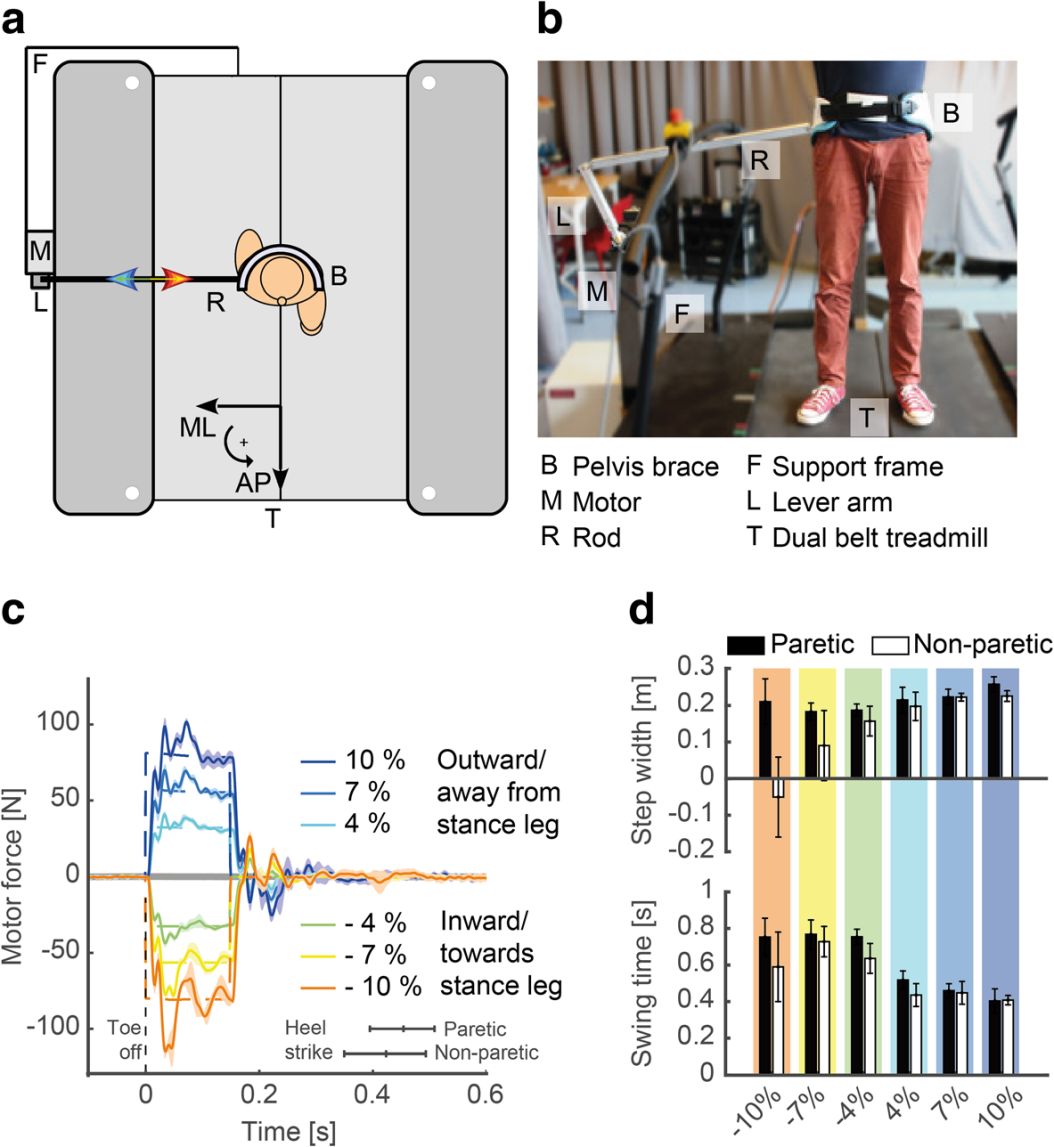
To systematically assess how patients maintain their balance when being perturbed during walking, the University of Twente has developed a pelvic perturbation device (**a** and **b**) [36]. This device consists of an admittance-controlled motor (Moog, Nieuw Vennep, the Netherlands) connected via a lever arm and a rod to a pelvic brace worn by the subject. The device allows providing perturbations in different directions, with different magnitudes and different durations at precisely timed instances of the gait cycle while walking on a treadmill (**c**, mediolateral perturbations timed at toe off with magnitudes expressed as % of body weight). In collaboration with Roessingh Research & Development, the device was used to assess the foot placement strategies of ambulatory stroke survivors when being perturbed away or towards the stance leg at the start of swing of the paretic or non-paretic legs. Responses of the step directly following the perturbation in a single stroke survivor are indicated in (**d**). Whereas the stroke survivor made a cross step, as evidenced by the negative step width, with his non-paretic leg when being forcefully perturbed towards the paretic leg, he did not make a cross step with his paretic leg. When being perturbed away from the stance leg, both the paretic and non-paretic side only slightly adjusted the step width but the foot was placed faster on the ground, as evidenced by the decreased swing time, to counteract the perturbation

**Fig. 3:**
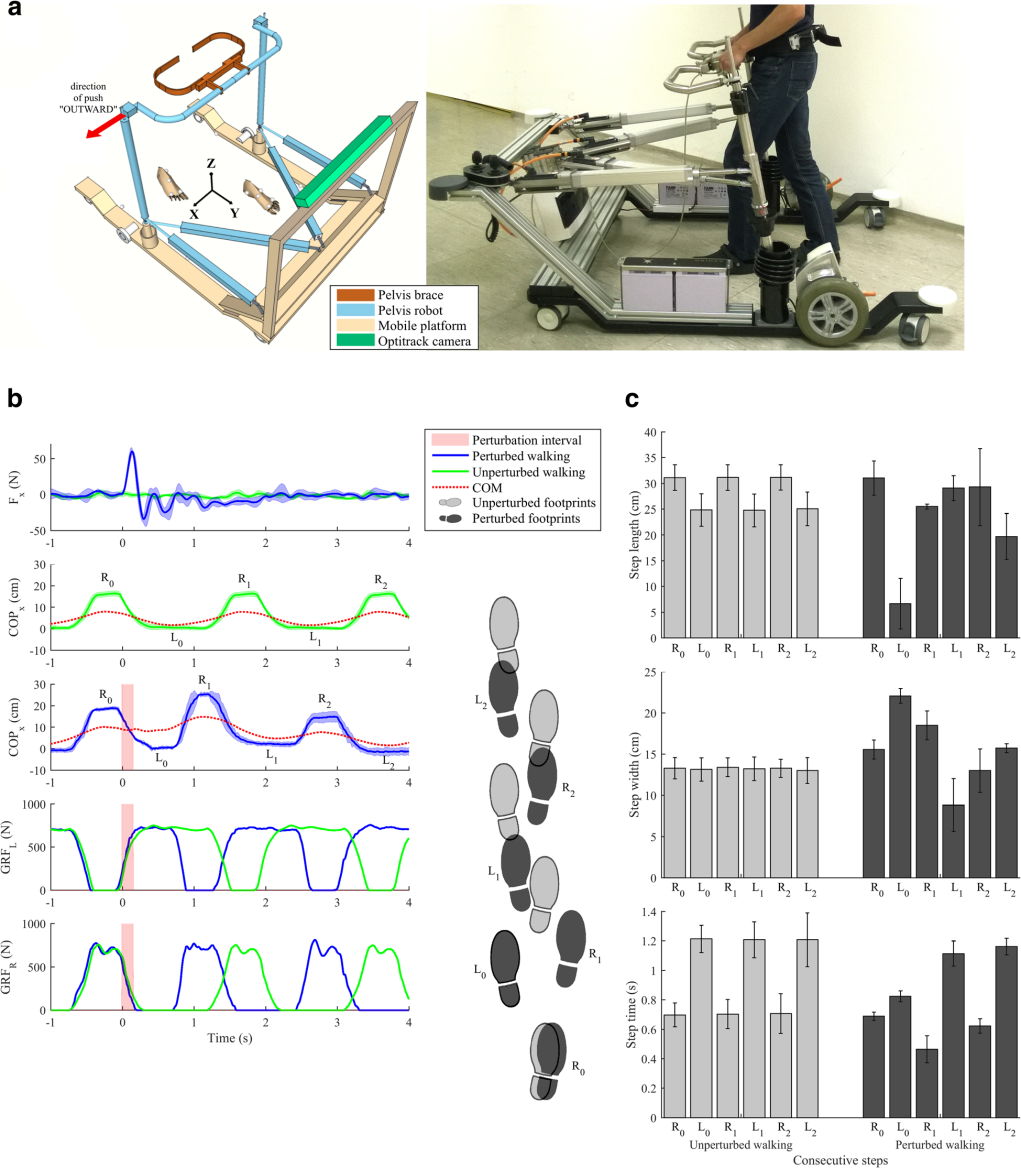
At the University rehabilitation institute, Ljubljana, Slovenia, we have developed a novel balance assessment robot (BAR). BAR is an admittance-controlled device that provides three actuated DOFs (sagittal and lateral pelvis displacements and rotation around vertical axis) while the remaining three DOFs (pelvic tilt, list and vertical displacement) are passive and spring loaded. BAR is placed on a mobile platform for over ground walking but can also be mounted onto an instrumented treadmill. Further details on BAR can be found in Olenšek et al. [37]. **a** shows schematics and a photograph of the actual system with indication of the “outward” perturbation direction. BAR can provide assistive force fields as well as apply perturbing pushes. In **b** a set of measurements are given illustrating unperturbed walking as well as balancing responses following a force impulse (50 N in duration of 150 ms) to a right-sided hemiparetic subject walking at 0.3 m/s (**b**-1). The push was delivered at the beginning of the stance phase of the non-impaired left leg and was directed to the right, i. e. »outward«. The applied push provoked movement of the COM to the right (**b**-3) as compared to unperturbed walking (**b**-2), the duration of the stance phase was significantly reduced (as indicated by the vertical GRFs – **b**-4 and 5) while the impaired right leg was placed more to the right (as compared to unperturbed walking) to enable adequate displacement of the COP in the lateral direction. **c** shows spatio-temporal characteristics of unperturbed and perturbed walking. The first bars in each graph sequence belong to a step that was completed prior to perturbation occurrence (from −1 s – 0 s) while the further five consecutive bars denote values for the steps following the commencement of the perturbation. Unperturbed walking is characterized by shorter steps that exhibit longer duration on the impaired side (right leg) compared to the unimpaired side (left leg). The perturbation is handled in the first step by substantially reduced step length and step time and increased step width of the first step. In the remaining steps, parameters gradually returned to those observed in unperturbed walking. All results show mean values and standard deviations of five individual trials. These results illustrate that well-defined perturbations and rather repeatable dynamic balancing responses can be obtained in neurologically impaired individuals. Thus, utilising the presented BAR robot to capture balancing responses in a form of COP, GRF, step lengths, step widths and step times before and after a therapeutic intervention can give objective assessment of each subject’s performance and efficacy of the applied rehabilitation

Robotic pusher devices are designed to provide controlled forces, such as pushes or pulls, to the pelvis or trunk during standing or treadmill walking. A device of this type is presented as example 1 (Fig. 2) [38], another example is the BAR-TM, similar to the device presented in Fig. 3 [39]. These robots are classified here as connector-, fixed-, treadmill-type (CFT) robots. Intrinsically, these devices measure the interaction force at, and the motion of, the single point of contact, which is generally closely related to the motion of the COM. More complex devices could potentially also support or correct pelvis motions, including support of the body weight.

#### Overhead active body weight support systems (CFT/CFP/CFO/CMO)

Body Weight Support (BWS) systems allow the generation of a constant or controlled vertical supportive force to provide safety and body-weight support during balance and gait training. Such devices are only robots if they have a controllable actuator, for example to follow the patient’s movements to provide a strictly vertical force, or to control the amount of BWS according to momentary need. Robotic BWS systems can support training on treadmills (in this case, classified as CFT) [40, 41], over-ground with a fixed mounting (in this case, classified as CFO) such as for example the ZeroG [42], over-ground on a mobile frame (CMO) [43], or potentially also on actuated footplate systems (in this case, classified as CFP).

The typical active BWS system is not able to provide horizontal perturbations, as required for advanced balance assessment, but more complex BWS systems, such as the FLOAT [44], may be used to provide a range of perturbations when standing [45]. However, it is impossible to transfer purely horizontal forces through cable-based systems like the FLOAT that have been developed to primarily provide controlled vertical forces. Additional robotic devices could be combined to provide perturbations.

In general, BWS systems are suitable to provide AAN support to ‘severely affected’ patients to stand or walk and thus facilitate their assessment.

#### Mobile self-balancing platforms for balance training (SMP)

Devices with a standing surface mounted on two wheels with an upright handgrip, that are able to self-balance on their two wheels, have been implemented as rehabilitation robots for balance training in patients with neurological disorders, for example the “Balance Training Assist” [46]. The standing surface tilts forward and backward, thus challenging the standing balance of the patient. These robots are classified as surface-, mobile-, platform-type (SMP) robots. With this configuration, they are only able to provide sagittal perturbations, either by rotating the standing surface around the wheels, or by accelerating forward or backward.

#### Mobile robotic gait trainers (CMO)

Mobile robotic gait trainers are robots that connect to the user at the pelvis, lower- or upper-trunk, while being mounted on a wheeled platform. They are used to provide body weight and/or posture support as well as safety during over ground gait and balance training. These devices can have various passive and actuated degrees of freedom, or, alternatively, spring-based posture correction [47, 48]. The robotic component can be the support system, when it controls the interaction force with the patient, or the wheeled base, when it provides automated navigation or actively follows the patient’s walking path.

These robots are classified as connector-, mobile-, over-ground-type (CMO) robots. Only devices with an active, controllable interface to the patient can provide controlled perturbations. A device of this type, e.g., as example 2 (Fig. 3), the BAR-OG, can apply pushes in various directions in the transverse plane, and also provide assistive forces as needed (AAN) to keep balance. Another example of this type is a wheeled platform that interfaces with the human upper body and allows variable support, resistance, and perturbations in all degrees of freedom during standing and walking on even ground, but also during more challenging activities such as stepping over obstacles and walking on uneven or soft terrain, i.e. the KineAssist [49].

#### Treadmill-mounted exoskeletons (DFT)

Treadmill-mounted exoskeletons are devices that allow actuating the user’s leg joints or segments through a set of robotic links. These robots are installed above a treadmill, such that the weight of the device is not supported by the patient. These robots are classified here as distributed-, fixed-, treadmill-type (DFT) robots.

Many of the first-generation of such devices constrained the pelvis in the coronal and sagittal planes, thereby ensuring standing/walking balance, which made them unsuitable for balance assessment. Recently developed devices allow or also actuate the pelvis’ translations in the medial-lateral and anterior-posterior directions and rotations in the transverse plane, in addition to allowing hip ab/adduction, such as LOPES I [50], LOPES II [51], the newer Lokomat [52], and other similar devices [53]. This allows patients in the device to maintain balance by themselves, and assessment of their balance control can be implemented. This type of device can provide perturbations as well as provide AAN.

#### Mobile-platform-mounted exoskeletons (DMO)

Mobile-platform-mounted exoskeletons are similar to type VI, with the exoskeleton mounted on a mobile wheeled platform that supports the weight of the device [54]. Exoskeletons that completely support their own weight through their foot segments are also under this type, as they are mobile by walking ability. An example of this type is the REX exoskeleton of Rex Bionics [55].

These robots are classified here as distributed-, mobile, over ground-type (DMO) robots. This type of device can provide perturbations as well as provide AAN, similar to type VI.

#### Fully wearable exoskeletons, actuated orthoses, or exo-suits (DWO)

Fully wearable exoskeletons, actuated (multi-joint) orthoses, and exo-suits are devices that actuate one or more leg joints of the user, similar to type V devices, but are fully body-worn [56]. These robots are classified here as distributed-, wearable-, over ground-type (DWO) robots.

Currently, the primary use of fully wearable exoskeletons is locomotor training or support of patients with a complete spinal cord injury [57]. Several of such devices are currently commercially available [55]. The implementation of the assessment of static balance on patients that are normally wheelchair-bound should be further explored, as these exoskeleton users are unlikely to stand or react to perturbations on their own, even though they could be capable of weak or diminished responses. Devices of this type are also proposed for stroke rehabilitation, where they can be used to provide perturbations as well as AAN for assessment, similar to type VI and VII devices.

Currently, most commercial exoskeletons are only able to perform assessment in the anterior-posterior direction because of their motion capabilities [55]. Assessment of balance in the frontal plane is not possible for current robots, since trying to tilt the exoskeleton to the left or the right poses a serious falling hazard due to lack of actuation and motion possibilities. In contrast to commercially available exoskeletons, some research exoskeletons, for instance the Mindwalker [58, 59], have actuation of hip ab/adduction, which allows assisting in weight-shifting and foot placement; this opens up the possibility for medial-lateral balance assessment. Fully wearable exoskeletons can support upright posture, but have no intrinsic BWS system, and stability and safety are usually provided by the use of crutches or an overhead BWS system. Since crutches alter the BOS, it is recommendable to carry out assessments using a BWS system without crutches. The actuated joints of the exoskeleton can be used to apply perturbations. Due to limited degrees–of-freedom, perturbations in the current generation of exoskeletons may only be applicable in the sagittal plane. This is a strong limitation, since perturbations in everyday life are not restricted to this plane.

#### Actuated foot plates, or ‘end-effector-connected robots’ (SFP)

Actuated foot plates, or ‘end-effector-connected robots’, refer to a type of robot that only connects to the user through the foot soles, and which actuates each foot separately. Each foot is connected to a haptic contact plate, which can emulate both stance and swing interaction (including other interactions such as slipping), and also support foot and leg movements, e.g., the Haptic Walker [60].

These robots are classified here as surface-, fixed-, plates-type (SFP) robots. Intrinsically, these devices measure the motions of the feet, which allows reconstruction of the BOS, as well the ground contact forces (or COP).

Depending on the device, perturbations can be provided in the anterior-posterior or medial-lateral directions, as well as their combination.

### Balance assessments performed with robots are device type-specific

The different types of rehabilitation robot have specific possibilities to provide assistance-as-needed (AAN), and/or to provide specific perturbations. How the robot interacts with the user directly determines what kind of perturbations can be implemented for assessment. For example, surface-type robots (S) can provide moving ground perturbations, connector-type robots (C) can provide push/pull perturbations, and distributed-type robots (D) can provide joint perturbations. This classification factor (1) is also related to the ability to assist-as-needed for assessment of ‘severely affected’ patients: surface-type robots (S) typically have no ability, connector-type robots (C) have some ability, for example to support body weight, and distributed-type robots (D) have the highest ability to support a patient, especially for complex tasks such as walking.

Table 3 indicates the suitability of each example type of device to provide specific types of perturbation, to be used for different assessment conditions, as well as their suitability for obtaining different measured parameters. The content of the table is determined by inspection of the capacities of the different example systems, such as: the ability to provide support to patients in standing or walking, and provide perturbations during these activities. Additionally, we considered whether there are limitations in such devices for performing the AAN or the perturbations, such as limited accelerations, limited degrees-of-freedom, or complexity of the robot. Therefore, with further technical developments, these characterizations could be modified to achieve specific goals.

**Table 3:** Suitability of different types of rehabilitation robots for assessment of balance in stroke patients. Classification is based on a robots’ potential ability to provide balance assessments and deliver perturbations to balance

Example robotic devices	Type of assessment Suitability			Type of perturbation Suitability			Quantitative measurements Suitability		
	Severely affected patients	Balance assessment in standing	Balance assessment in walking	Moving ground	Horizontal Pushes	Joint	Embedded sensors	Wearable sensors	External sensors
Perturbation platform (SFP)	−/+^a^	+	−	+	−	−	GRF SK	GRF BK	BK SK
Robotic pusher devices (CFT)	+	+	+	−	+	−	CIF CK	GRF BK	GRF BK CK
Active Body Weight Support systems (CFT/CFP/CFO/CMO)	+	+	+	−	+	−	CIF CK	GRF BK	GRF BK CK
		Robotic BWS systems typically provide AAN, but can be combined with other robotic devices for providing perturbations.							
Mobile self-balancing platforms for balance training (SMP)	−/+^a^	+	−	+	−	−	GRF SK	GRF BK	GRF BK SK
Mobile robotic gait trainers (CMO)	+	+	+	−	+	−	CIF CK	GRF BK	GRF BK CK
Treadmill-mounted exoskeletons (DFT)	+	+	+	+	+	+	GRF CK (CIF)	GRF BK	GRF
Mobile-platform-mounted exoskeletons (DMO)	+	+	+	−	+	+	CK (CIF)	GRF BK	GRF
Fully wearable exoskeletons, actuated orthoses, or exo-suits (CWO)	+	+	+	−	−	+	CK CIF	GRF BK	GRF
Actuated foot plates, or ‘end-effector foot-connected robots’ (SFP)	+	+	+	+	−	−	CK CIF	(CIF) BK	BK

*SK* Surface Kinematics: Inclination or translation from the center of standing surface (position, speed, acceleration), *BK* Body Kinematics: COM/Sacrum, configuration of a segmental representation of the body (position, speed, acceleration), *CK* Connection point (s) kinematics: Points where the robotic device is connected to the body (position, speed, acceleration), *CIF* Connection point (s) Interaction Forces: Points where the robotic device is connected (6D, 3D, 1D force or pressure distribution), *GRF,* Ground Reaction Forces: Contact between foot and standing surface (6D-, 3D-, 1D–force or pressure distribution)

^a^ Depending on exact configuration

- unsuitable; + suitable

To quantify the performance and reactions of a patient under AAN and perturbations, different metrics could be used. Such metrics, paralleling those in posturography, will typically contain information on the movement of the COM, COMv or XCOM, and of the BOS or COP. The sensors embedded in the robot for its actuation and control could be sufficient to estimate such parameters, but typically additional sensors have to be added. These sensors can be wearable, or also have fixed components in the environment.

Kinematic quantities that are typically measured are the kinematics of the standing surface, the configuration of a segmental representation of the human body or the connection point (s) of the robotic device to the body. Kinetic quantities that are typically measured are the forces at the connection point (s) of the device to the body and the ground interaction forces. Interaction forces can be measured as force in the main direction of interaction (1D), as all force components (3D) or also including the torques (6D); related information can be obtained by measuring the pressure distribution at the surface of interaction.

Instruments or sensors that are relevant in this context are force plates, wearable (e.g., IMU-based) or camera/marker-based motion capture systems, force shoes [61], pressure insoles, as well as all robot-embedded sensors for movement and force measurements.

How the robot interacts with the patient (classification factor 1) and on which surface the robot is operated (classification factor 3) will limit the need for (and feasibility of) combination with different additional measurement systems required for specific assessment metrics. Surface-type robots (S) often intrinsically measure ground reaction forces, and may be combined with fixed or wearable (COM-) motion capture systems; connector-type robots (C) at most intrinsically measure pelvic movement, which can be used to determine COM, but may require motion capture systems and force plates or pressure insoles to determine kinetic parameters; and distributed-type robots (D) typically measure joint motions, which allow for reconstructing body or COM motions, but may require force plates or pressure insoles or ground contact sensors to determine COP- and BOS-related information.

Robots that are operated overground (O) can be best combined with wearable or mobile measurement systems for motion capture or ground interaction measurements; robots that are operated on a treadmill (T) and robots that are operated through plates (P) are more easily combined with fixed measurement systems, such as fixed motion capture or force plates, or have treadmill-integrated force measurement.

### Metrics in robotic balance assessment need device type-specific adaptation

To use robots for assessment, quantitative validated metrics should be available to measure performance. Related to the specific properties of the robots, these metrics should reflect: a) how much assistance (or resistance) is provided to the patient during task execution, and/or b) how the patient reacts to applied perturbations. Metrics that contain such information inherently allow monitoring the improvement or deterioration of balance control over time.

The quantification and measurement of the amount of support depends on the type of robot being used, and needs to be specific to the system. Therefore, there is a need for methods and standardized procedures on how to ‘instruct’ robots to provide just the amount of support needed for the patient to perform the function, as well as a metric to quantify the amount of support in that specific type of device. This is directly related to the concept of “Assistance-As-Needed” (AAN) that is used as a training approach [31]. An example is the critical percentage of Body Weight Support (indicated as a percentage of full body weight) that is required for a subject to stand or to walk (although this reflects several body functions and not just balance).

The reaction of a subject to a perturbation can be quantified by methods and metrics that have been developed in posturography and gait analysis, together with extensions towards generalized perturbation analysis. A review of posturographic methods and metrics can be found in [62]. An overview of regularly used metrics is provided as Appendix to this paper. These metrics typically contain information on the kinematics of body motion, especially movement of the total body or its main segments (more precisely, of their COM), as well as on the body’s interaction with the ground, e.g., through ground reaction forces, base of support or COP (for definitions refer to Fig. 1, and for metrics refer to the Appendix).

In scientific practice, marker-based motion capture systems and force plates are the reference tools to measure COMs and COPs, but both are expensive, bound to a fixed location, require a lot of time to set up, calibrate and post-process, and may be problematic in an environment loaded with different reflective or magnetic equipment, i.e., around robots. Costs could be better justified in robotic devices used both for assessment and therapy. Simpler procedures may be adequate for clinical assessment, as opposed to scientific measurement. For instance, single IMUs attached to the sacrum of healthy subjects provide fairly accurate estimates of the COM movement during walking, and shoes instrumented with force sensors, IMUs and ultrasound sensors adequately estimate relevant quantities like the COM, COP and BOS in healthy and stroke gait [23, 63, 64]. Low-cost consumer motion sensing devices also hold a promise for adequate estimation of body segment kinematics from advanced processing algorithms, which can be used to obtain adequate COM estimates [65, 66]. Different types of robots also intrinsically measure quantities related to human motion kinetics, and such information may very well be used to obtain adequate estimates of the relevant kinematic quantities in order to calculate device-specific metrics.

Considering the huge difference between concepts and implementations of the different robots, it is necessary to select device-specific metrics, as well as implement and validate a reliable acquisition of these metrics. Preferably, the clinical user community of a specific device should, together with the developer, evaluate which metric is best suitable for a specific device, given its technical feasibility as well as clinical value.

### Normative assessment scores need to be device- and assessment procedure-specific

Similar to current clinical and research balance assessments, it is critical to establish normative values for robotic-based assessments to better interpret and use them. Normative scores in assessment measures are generally measured with the exact same procedure, device, and robot settings, in a representative (impaired or age-matched unimpaired) subject group. For this reason, generic and device-unspecific datasets are hardly useful towards the composition of normative scores. Although many studies use comparable metrics, it is important to be aware that measures can probably only be compared when using the same perturbation device (considering the diverse configurations presented presented above, see Table 2), the same kind and amount of support, and with the same procedures, environment and perturbations. For this reason, reference values of assessments are best provided by the device manufacturers or end-users (e.g., the clinical community). When developing new robotic devices for balance assessment, studies are needed to establish databases of normative values relative to each particular assessment method. This indication of reference normality is important to detect specific abnormalities. Linking such identified components of poor balance performance to specific effective training modalities is a next, largely open field of clinical research. The body of knowledge from the field of posturography should be used as a starting point and a reference. To only measure the progress within a specific user, detailed comparison to an able-bodied reference group may not be needed.

### Examples of balance assessments using robotic devices

In Figs. 2 and 3, we provide two illustrative examples of robotic devices that are used for assessment of post-stroke subjects (in research). Both provide quantitative information on balance performance through measuring the reaction to perturbations. The robotic devices as well as illustrative assessment results with one patient are presented. These robots have been developed to perform research to understand normal and impaired human balance, which is related to assessment. In both cases, different perturbations and different metrics were used to perform the assessment, according to the capabilities of the robot, as well as to illustrate how different metrics can provide comparable information. In the classification described above, these example assessments classify as, for example I, type II - treadmill-mounted pusher device (CFT), and for example II, type V - mobile robotic gait trainer (CMO).

Another example rehabilitation robot used for assessment is the Kine-Assist, a device that was initially developed as a type V - mobile robotic gait trainer (CMO) [49], but is also used in an adapted version fixed over a treadmill (CFT). A device-specific assessment procedure was developed and validated, called ‘Kine-Assist 9 Task Balance Test (K-9)’ [67].

## Conclusion and outlook

We have presented an overview of current balance assessment procedures in clinical practice and in research. Based on this overview, we evaluated the potential use of rehabilitation robots as tools for such assessment. The main benefits of using robots for assessment are the possibility to assess ‘severely affected’ patients by providing assistance-as-needed (AAN), as well as providing consistent perturbations during standing and walking while measuring the patient’s reactions, thus creating an important extension to ‘classical’ posturography. We provided a classification of rehabilitation robots in three aspects, relevant to their potential application for assessment. Nine sample types of state-of-the-art rehabilitation robots were described in more detail and evaluated for their suitability for balance assessment. Two example cases of robotic assessments that apply perturbations during walking were presented to illustrate the new possibilities.

We believe that rehabilitation robots are promising, and can become useful and relevant tools for assessment of balance in patients with neurological disorders, both in research and in clinical use. Once their potential for improved assessment is realized, robotic assessments may provide sufficient information to allow individual tailoring of training, which may largely improve training effectiveness. The two examples provided in this paper already illustrate that robotic methods may reveal specific deficiencies underlying poor balance performance, which can be targeted in specific, individualized training approaches. In order to realize the potential to improve assessment and therapy of balance, several improvements have to be made to current robotic devices, and further research is needed on methods of application for assessment. Important considerations in this respect are:

- Use for balance assessment should be taken into account when developing new rehabilitation robots, especially in the aspects of: quantification of support (AAN), perturbations provided in a transparent control mode (allowing unhindered subject responses), and sensors to collect relevant data.
- Simplified systems, compared to currently commercially available rehabilitation robots, could already provide highly useful tools for assessment and training. This could also make systems more affordable for clinical practice.
- The user community of a specific device should, together with the developer, evaluate which metric is best suitable for a specific device, such that it is technically feasible as well as clinically useful. Consensus across the field for all devices will be impossible to achieve, considering the lack of consensus on which metric to use, as well as the technical differences among robotic devices and measurement systems. Such consensus may follow the increased use of robotic devices for assessment in research and clinical practice.
- For each rehabilitation robot that will be used for balance assessment, normative reference data should be collected with unimpaired subjects.
- Better understanding of human balance control and its underlying functions and mechanisms will enable improved design of assessment methods, improved implementation of robotic assessments, as well as comparability of results obtained with different rehabilitation robots or assessment procedures.
- Better understanding of human balance control should lead to convergence of the multitude of outcome measures to a core set of essential metrics that then can be used to define a universal balance assessment set of metrics.
- Once a core set of methods and metrics is defined, these metrics should be validated as assessment metrics by performing longitudinal studies to establish their validity and sensitivity.
- Ahead of a generally agreed robotic assessment method, device-specific assessment methods can be used to monitor progress of individual patients in parallel to established clinical metrics.
- Each individual suffering from neurological damage is a case on its own, despite common aspects in functional limitations. In order to tailor training to the individual needs, adequate functional diagnostics is required.
- Research is needed on how in detail such functional diagnostic information can be used to optimize outcome results of rehabilitation training for the individual cases.
- The more complex the robotic device configuration (e.g., exoskeleton compared to perturbation platform) the more complex it will be to minimally interfere with the subjects’ reactions. Realistically, in order to perform proper assessment of balance through wearable robots like exoskeletons, substantial technical improvement on the state-of-the-art is required, mostly in order to allow unhindered movement of the patient, both with respect to degrees of freedom as well as added inertia and general transparency.
- The volume of soft tissue between a robotic device and the skeleton can have a considerable effect on the accuracy and repeatability of the perturbations that the device can provide as well as the obtained sensor information, and must be carefully considered.

## Appendix

### Posturographic metrics related to assessment of dynamic balance

{this information is thought to be useful to some readers, but not of sufficient importance for the main text, hence we included it as an Appendix}

#### Posturographic metrics

Table 4 provides an overview of common metrics from posturography and gait analysis used to quantify aspects of balance. All these metrics contain rich information related to balance performance in standing or walking, and can, in principle, be used to quantify a response to a perturbation during walking. Currently, there is no scientific consensus on the most suitable metrics for assessment through posturography, which factors should determine the optimal choice of metrics, or how to interpret different outcomes and relate them to functional limitations. Different posturographic metrics are likely to quantify different aspects of balance, and thus may be complementary.

**Table 4:** Review of metrics used to quantify balance performance during standing and walking, usable for robotic assessment

Definition of metric	Suitability for Walking or Standing	References
AREA of COP or COM motion (e.g., 95% confidence circle or ellipse area)	Standing	[17, 86–88]
PATH LENGTH of COP or COM motion	Standing	[86, 87]
STATISTICS of COP or COM motion (e.g., centroid, x^th^ percentile, median, dispersion)	Standing	[17]
DISPLACEMENT of COP or COM (e.g., RMS, maximum excursion, range, ratio of AP/ML)	Standing and Walking	Based on COP [17, 19, 89–91] Based on COM [92]
RELATIVE MOTION of COM relative to COP; COM to BOS; COP to BOS (e.g euclidian distance, time-to-contact)	Standing and Walking	[93–97]
SYMMETRY of COP or COM motion (e.g., left-right ratio)	Standing and Walking	[98–102]
LONG-TERM CORRELATION in COP or COM motions (e.g., Hurst exponent, Largest Lyapunov Exponent)	Standing and Walking	[103–108]
VELOCITY of COP or COM (e.g., average, range)	Standing and Walking	[17, 19, 86, 88, 90, 109]
TOTAL BODY ORIENTATION (e.g., range of angle or angular velocity)	Standing and Walking	[89]
JOINT KINEMATICS - angle or angular velocity of joints (e.g., RMS, maximum excursion, range, time series)	Standing and Walking	[110]
JOINT KINETICS - joint torque, torque rate of change (e.g., RMS, maximum excursion, range, time series)	Standing and Walking	[76]
TEMPORAL-SPATIAL gait parameters (i.e., step length, stride length, step width, swing duration, temporal stance-swing ratio)	Walking	[76]

The table lists metrics that quantify balance, as used in posturography and gait analysis. Most of the presented metrics have been validated by showing that they are significantly different among groups or conditions with different balance behaviour such as elderly versus young adults, or eyes opened versus eyes closed condition. Readers are referred to the indicated studies for approaches or methods for calculation and for detailed information about which groups or conditions the methods have been shown to be indicative.

These metrics are all applicable to in assessment of all three types of balance control: steady state, anticipatory, and reactive. It can be expected that descriptive statistics e.g., average, minimum, or maximum of these metrics are highly influenced by the assessment procedure, especially during reactive balance, when reacting to device specific perturbations.

Many of the metrics of Table 4 have been shown to correlate with risk of falling or with one or more of the clinical scales of Table 1, or they are significantly different between groups or conditions with different levels of balance performance such as elderly versus young adults, stroke subjects versus unimpaired age-matched controls, or eyes opened versus eyes closed conditions. Readers are referred to the indicated studies for approaches or methods for calculation and validation studies.

The exact understanding of normality and abnormality of the values of metrics in posturography remain a topic of research [19]. The metrics generally show inter- and intra-subject variability due to use of different movement strategies depending on age, disease, test condition and testing equipment [19, 68–70], preventing straight-forward comparisons of individual assessments with reference performance values.

#### Assessment of dynamic balance

Dynamic, as opposed to static, posturography includes reactions to external perturbations applied by movements of the standing surface, and is related to ‘reactive balance control’, and therefore of specific interest for robotic assessments. For dynamic conditions in general, including walking, the static balance requirement of keeping the COMv in the BOS is inadequate, as the COMv may temporarily leave the BOS as long as it returns before a fall occurs. Thus, not only the position of the COM-projection but also its velocity and acceleration determine whether or not it is possible to maintain balance without taking a step or generating inertial moments with the upper body. To quantify this, the “extrapolated center of mass” or XCOM was defined [71, 72]; when the vertical projection of the XCOM does not leave the BOS, balance can be maintained without making a step. For perturbations where the XCOM cannot be brought back inside the actual BOS, a fall may still be prevented by making an adequate step, the stepping strategy, i.e., by adjusting the BOS so that the COMv can still return into it. To properly interpret the motions of COP and (X) COM in their inter-connection, methods applying well-defined perturbations (external, sensory or motor perturbations) in combination with closed-loop system identification techniques are currently explored in research [18, 73]. Such techniques may either reveal causes of abnormal COP and COM motion and control, or identify which of the underlying systems (e.g., vestibular system, muscle force generation, muscle coordination) are deteriorated, and to which degree [18, 74].

The same principles apply when maintaining balance during a dynamic task like walking, where balance can be understood as ensuring that the XCOM does not diverge too far away from the BOS and can always be brought back towards and/or inside the BOS. This approach has been described under the name ‘capture point’ in robotics [75]. Gait analysis, as performed in specialized gait labs, analyses (ab-) normality of walking, but typically only by comparing spatiotemporal parameters and patterns in kinematic variables, but not by analysing posturographic parameters or patterns based on formal balance requirements, nor through measuring reactions to perturbations [76].

Nevertheless, the quantities measured in gait analysis can be used to quantify reactions to perturbations. For walking, especially when it comes to assessing the response to perturbations in the walking direction, it is important to consider ‘healthy strategies’, relate them to concepts like XCOM and capture point, and study what type of deviations from normality occur in patients. If such patterns are identified, critical metrics may be related to them. Initial work in this direction can be found in [47].

## Abbreviations

AP: Anterior-posterior: forward and backward body motion
BBT: Berg balance test
BESTest: Balance evaluation systems test
BOS: Base of support
BWS: Body weight support
COM: Center of mass
COMv: Vertical ground projection of the center of mass
COP: Center of pressure
DOF: Degree of freedom
FLOAT: Free levitation for overground active training, an overhead BWS system
IMU: Inertial measurement unit
ML: Medial-lateral: left and right body motion
ODR: OmniDirectional Treadmill
POMA: Performance-oriented mobility assessment
STARS: STate of the Art Robot-Supported assessments
TUG: Timed up and go
